# A Diagnostic Surprise: Primary Hodgkin’s Lymphoma of the Lung

**DOI:** 10.1177/2324709617734247

**Published:** 2017-10-03

**Authors:** Ankur Sinha, Ravikaran Patti, Prabhsimranjot Singh, William Solomon, Yizhak Kupfer

**Affiliations:** 1 Maimonides Medical Center, Brooklyn, NY, USA

**Keywords:** Hodgkin’s lymphoma, primary lung tumor

## Abstract

An 81-year-old male presented to the emergency room with a 3-month history of progressive shortness of breath, productive cough with white sputum, and generalized weakness with 10-pound weight loss in 2 months. On presentation, the patient was afebrile, with blood pressure of 93/55 mm Hg and oxy-hemoglobin saturation of 92% on 2 liters of oxygen via nasal cannula. Complete blood count with differential was significant for white count of 12 400/mL. Brain natriuretic peptide level was 454 ng/mL. Postero-anterior chest radiograph showed multiple round opacities in the lung fields. Computed tomography scan of the chest confirmed multiple round densities in both the lung fields along with mild mediastinal lymphadenopathy. Core needle biopsy was performed. Immunohistochemical stains were positive for CD30 and CD15 in a population of large atypical cells amid a background of CD3-positive nonneoplastic cells. These results were in support of the diagnosis of classical Hodgkin’s lymphoma of the lung with histological appearance confirming nodular sclerosis type. The patient was started on chemotherapy but was readmitted in 20 days for acute respiratory distress and suffered cardiac arrest and subsequently died. This case highlights the fact that although primary pulmonary Hodgkin’s lymphoma of the lung is a rare entity, it should be thought of as a differential while evaluating lung masses. In these cases, definite diagnosis can only be made by biopsy and histology. Early commencement of chemotherapy and regular follow-up with oncology is essential.

## Introduction

Primary pulmonary Hodgkin’s lymphoma (PPHL) is exceedingly rare with fewer than 100 cases reported worldwide. Radin initially reported a review of worldwide literature in 1990, with about 61 cases between 1927 and 1990.^1^ PPHL is caused by a clonal proliferation of the lymphoid cell line, forming a mass in the lung. We report a rare case of an 81-year-old patient with PPHL. The rarity of this disease, combined with a lack of randomized clinical trials for therapy, warrant vigilant reporting of case presentation as well as clinical outcomes.

## Case Presentation

An 81-year-old male with a history of hypertension, atrial fibrillation, congestive heart failure (CHF) with ejection fraction of 45% presented to the emergency room with progressive shortness of breath for 3 months. He also complained of cough, productive of white sputum with generalized weakness and 10-lb weight loss over the past 2 months. On presentation, the patient was afebrile, with blood pressure of 93/55 mm Hg, and oxy-hemoglobin saturation of 92% on 2 liters of oxygen via nasal cannula. Physical examination revealed crackles over bilateral chest, 1+ lower extremity edema bilaterally. Blood tests showed white count of 12 400. Brain natriuretic peptide level was 454 ng/mL. Cardiac troponin T was negative.

**Figure 1.**
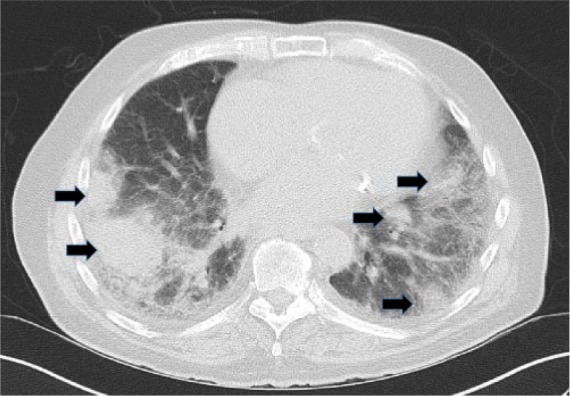
Computed tomogram of the chest, axial cuts with arrows pointing at the multiple nodular densities through the lung fields bilaterally.

Postero-anterior chest radiograph revealed round opacity in the right lung. Computed tomography scan of the chest was performed, which confirmed multiple round densities in both the lung fields (Figure 1) along with mild mediastinal lymphadenopathy. The patient was being admitted for treatment with acute systolic CHF exacerbation and was also being worked up for lung malignancy. The patient received diuretics that improved the breathing status.

Differential diagnoses at this stage were non–small cell carcinoma of the lung, adenocarcinoma of the lung, or lymphoma due to the presence of the lymphadenopathy. The patient underwent interventional radiology guided core needle biopsy for further evaluation of the lung densities. Immunohistochemical stains were positive for CD30 and CD15 in a population of large atypical cells amid a background of CD3-positive nonneoplastic cells. Stains for CD20 and CD1A were negative. These results combined with the histological findings support the diagnosis of classical Hodgkin’s lymphoma of the lung with histological appearance confirming nodular sclerosis type.

Due to extensive cardiac disease (CHF, atrial fibrillation, and bioprosthetic aortic valve replacement), the patient could not be started on standard chemotherapy with adriamycin, bleomycin, vinblastine, and dacarbazine. Instead the patient was started on cyclophosphamide, vincristine, and prednisone. The patient was discharged after medical optimization with follow-up at the cancer center, where he received a cycle of chemotherapy.

The patient was readmitted within 20 days for acute respiratory distress with hemodynamic instability. He was intubated and placed on vasopressor support. Clinical and radiological findings suggested acute respiratory distress syndrome. The patient subsequently suffered from a cardiac arrest and could not be resuscitated and expired.

## Discussion

PPHL is a rare disease with very few cases reported worldwide. PPHL is diagnosed with the aid of the following criteria: (1) features of Hodgkin’s lymphoma on histology, (2) disease limited to the lung with or without minimal hilar lymphadenopathy, and (3) exclusion of disease at distant sites.^2^ The commonest variety of this disease is the nodular sclerosis variant that comprises about 70% of PPHL.^3^ PPHL affects women more frequently than men.^1^

Clinical features of the disease are usually nonspecific, presenting mainly as difficulty in breathing, night sweat, long-standing cough with generalized weakness, and weight loss. PPHL may present as a solitary mass or as multiple nodular lesions. These lesions can frequently cavitate.^1^

**Figure 2.**
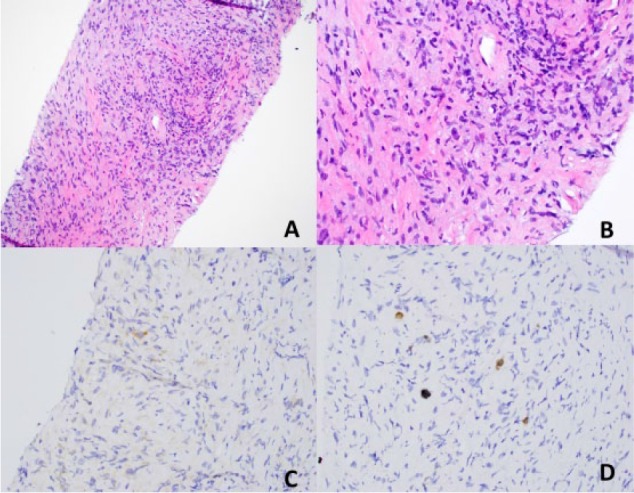
(A) Morphological features of tumor cells on low power (10×) showing ample RS cells. (B) High-power view of the tumor cells showing RS cells as well as variants. (C) Image showing CD30-positive cells. (D) Image showing PAX-5 gene product positive staining.

Previously Ann Arbor classification was used for pulmonary lymphoma staging.^4^ But the clinical presentation is very nonspecific. Sputum cytology may show Reed-Sternberg cells,^5^ although tissue biopsy is frequently needed. Needle biopsy with immunohistochemical staining is usually diagnostic. Specific morphologic features include dispersed neoplastic components, and Reed-Sternberg cells are universally present. Molecular characteristics include CD30, CD15, and PAX-5 gene product positivity. About 10% tumors may be CD3 positive. PAX-5 gene product positivity is important in differentiating classical Hodgkin’s lymphoma from anaplastic large cell lymphoma Hodgkin’s like (ALCL-HL).^6^ Our patient had Reed-Sternberg cells on microscopy. Tumor cells showed CD30, CD15, and PAX-5 gene product positivity. This confirmed the diagnosis of classical Hodgkin’s disease (Figure 2).

Traditionally in cases reported prior to 1960, the modality of treatment was surgical excision. Since then a better understanding of the pathogenesis of lymphoma has led to a preference for combination chemotherapy, especially for disseminated disease throughout the lung. Some reports have suggested the use of radiation therapy for limited disease, but the risk of radiation-induced pneumonitis should be considered. Prognosis of the disease depends on the presence of systemic symptoms (B symptoms), single versus multiple lesions, local and widespread invasion and cavitation.^1^

## Conclusion

PPHL is a rare disease, and it is difficult to diagnose due to its nonspecific presentation. Tissue biopsy for diagnosis and treatment with combination chemotherapy is key toward improving survival.
